# Micronutrient Deficiency in Children and Adolescents with Obesity—A Narrative Review

**DOI:** 10.3390/children10040695

**Published:** 2023-04-07

**Authors:** Valeria Calcaterra, Elvira Verduci, Chiara Milanta, Marta Agostinelli, Carolina Federica Todisco, Federica Bona, Jonabel Dolor, Alice La Mendola, Martina Tosi, Gianvincenzo Zuccotti

**Affiliations:** 1 Pediatrics and Adolescentology Unit, Department of Internal Medicine, University of Pavia, 27100 Pavia, Italy; 2 Pediatric Department, Buzzi Children’s Hospital, 20154 Milan, Italy; 3 Department of Health Sciences, University of Milan, 20142 Milan, Italy; 4 Department of Biomedical and Clinical Science, University of Milan, 20157 Milan, Italy

**Keywords:** pediatric obesity, micronutrients, children, deficiency, malnutrition

## Abstract

Childhood obesity represents a serious public health burden. Despite excessive dietary consumption, children with obesity present high rates of micronutrient deficiencies, such as deficiencies in minerals and specific vitamins; micronutrient deficiencies may have a pathogenic role in obesity-related metabolic comorbidities. In this narrative review, we analyzed the main deficiencies associated with obesity, their clinical consequences, and the evidence about a possible supplementation. Iron; vitamins A, B, C, D, and E; folic acid; zinc; and copper deficiencies represent the most common deficient microelements. The relationship between obesity and multiple micronutrient deficiencies remains unclear, and different mechanisms have been proposed. The medical care plan for pediatric obesity should include food choices with high nutritional content as part of a crucial approach to obesity-related complications. Unfortunately, only a few studies are available regarding the efficacy of oral supplementation or weight loss for treating them; thus, continuous nutritional monitoring is necessary.

## 1. Introduction

Childhood obesity has become a global epidemic and a serious public health burden; “globesity” is a new neologism that clearly describes this high-prevalence risk worldwide [1,2]. Indeed, the prevalence of worldwide obesity has nearly tripled from 1975 to 2016 in children and adolescents aged 5–19 years, from just 4% to 18%, respectively, reaching over 340 million children with obesity or overweight in this age group, similarly among both boys and girls [3]. Moreover, according to estimates by the World Health Organization (WHO), 39 million children younger than 5 years were obese or overweight in 2020, and the fact that obesity affects children as young as 2 years old is even more concerning [1]. A further increase in pediatric obesity has been associated with the recent COVID-19 pandemic [4,5]. If current trends continue, obesity is expected to affect 60% of adult men, 50% of adult women, and 25% of children by 2050 [1]. Prevailing evidence suggests that the rate of youth obesity has continued to increase, especially among children and adolescents with parental obesity [6].

Pediatric obesity is a crucial risk factor for adverse nutritional complications. The nutritional status of children and adolescents with obesity seems to be a paradoxical malnutrition. In fact, despite excessive dietary consumption, children with obesity present high rates of micronutrient deficiencies, such as deficiencies in minerals and specific vitamins; micronutrient deficiencies may have a pathogenic role in obesity-related metabolic comorbidities [7,8,9,10,11,12].

In this narrative review [13], we analyzed and summarized the main deficiencies associated with obesity, their clinical consequences, and the evidence about a possible supplementation. The medical care plan for pediatric obesity should include food choices with high nutritional content as part of a crucial approach to obesity-related complications.

## 2. Methods

An analysis of the available literature and a non-systematic summation on the topic of micronutrient deficiency in children and adolescents with obesity were proposed [13]. English-language relevant literature from the past 15 years was considered, including original papers, meta-analyses, clinical trials, and reviews. Letters, case reports, or series were not included. The abstracts of the literature were assessed (n = 128), and the full texts of potentially relevant articles (n = 45) were reviewed and analyzed by the authors. The reference list of all articles was also checked to identify relevant papers. The research terms adopted, alone and/or combined, were obesity, adolescents, children, micronutrient deficiency, trace element deficiency, mineral deficiency, deficiencies diagnosis, oral supplementation, nutritional intervention, iron, anemia, inflammatory anemia, vitamin A, vitamin C, vitamin D, vitamin E, vitamin B12, folic acid, zinc, and copper. The databases PubMed, Scopus, and Web of Science were used for the literature research. The contributions were independently collected by C.M., A.M., F.B., C.F.T., and J.D., and critically discussed and analyzed with V.C. and E.V. The resulting draft was critically revised by V.C., E.V., and G.Z. The final version was approved by all.

## 3. Childhood Obesity

Obesity is a pathological condition characterized by excessive adipose tissue storage as a consequence of an energy imbalance between caloric intake and expenditure [14].

The weight-to-length ratio is used to diagnose overweight and obesity in children up to 24 months old, using the WHO 2006 reference curves. After the age of 2 years, it is based on the body mass index (BMI), using the WHO 2006 reference system up to 5 years and the WHO 2007 reference system thereafter [15] (Table 1).

The underlying etiology of obesity is extremely complex and has been the subject of extensive research [1]. Childhood obesity is the result of a complex interaction among several risk factors involving genetic, environmental, nutritional, and socio-economic influences [15]. Although the pathophysiology of obesity remains controversial, the energy imbalance between consumed and expended calories is considered one of the main drivers of the development of obesity and overweight [16,17]. The WHO attributes the increased prevalence of childhood obesity to a global shift in diets towards energy-dense, nutrient-poor foods and increased use of sugar-sweetened beverages that greatly impact the risk of obesity due to high caloric intake [1]. These changes have also been accompanied by factors predisposing to chronic low energy expenditure, such as less physical activity due to increasingly sedentary lifestyles [15]. However, there are also socio-economic factors [18] as well as developmental factors such as genetic and epigenetic conditions that predispose to the risk of obesity [19].

The inflammation induced by obesity and associated behaviors can cause many correlated diseases [20]. Complications of obesity can occur in both the short and long term. As the prevalence and severity of pediatric obesity increases, these changes are also beginning to be seen in children [21,22,23]. Obesity is associated with an increased risk of serious health conditions including cardiovascular disease, high blood pressure/hypertension, renal comorbidities, gastrointestinal disease such as non-alcoholic fatty liver disease, non-alcoholic steatohepatitis, cholelithiasis, hepatocellular carcinoma, pulmonary disease such as asthma, and obstructive sleep apnea. Obesity is also related to endocrine and metabolic disorders such as dyslipidemia, hyperandrogenemia, early pubertal development, polycystic ovarian syndrome (PCOS), hyperglycemia, insulin resistance that can evolve into prediabetes and subsequently into type-2 diabetes mellitus, and musculoskeletal comorbidities such as foot and lower limb pain [2,4,15,24]. The main possible complications of obesity are illustrated.

Moreover, obesity is also often related to psychosocial complications; indeed, children with obesity are more likely to experience low self-esteem, develop depression, have a reduced quality of life, and experience social isolation during adolescence [8,9].

**Figure 1:**
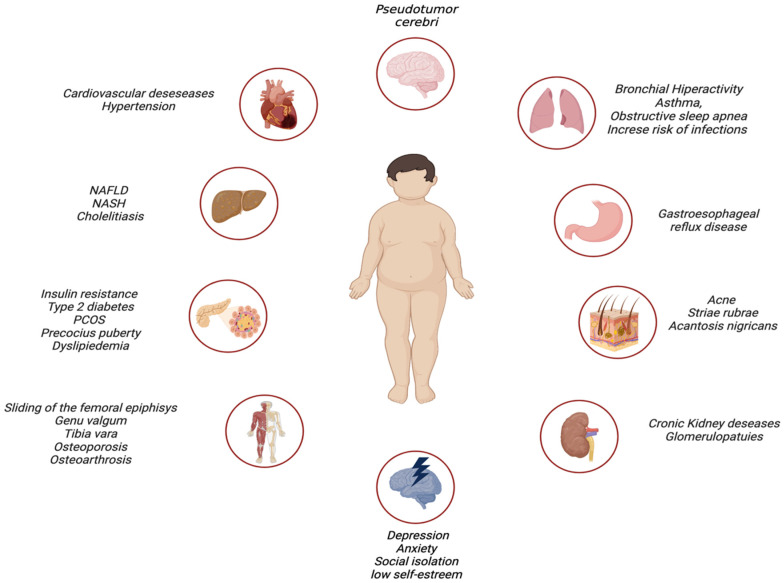
Comorbidities of obesity (created with BioRender.com (accessed on 15 March 2023)).

All these comorbidities contribute to increased morbidity and mortality in adulthood associated with being in the BMI range of obesity in pediatric age [6,7,8,9,10,11,12,13,14,15,16,17,18,19,20,21,22,23,24,25,26,27,28,29], Figure 1.

Since obesity involves important comorbidities both physically and psychologically, timely treatment of children and adolescents with overweight/obesity is extremely important. The treatment approach for childhood obesity should be broad, sequential, dynamic, and multidisciplinary [24,29,30].

Non-pharmacological preventive approaches and therapies are the most important treatments for childhood obesity. Firstly, breastfeeding is the primary prevention for overweight and obesity; a few studies reported a significative risk reduction (26%) of developing obesity later in life in breastfed children compared to those who received formula [24,31]. Additionally, an interesting systematic meta-analysis confirmed the protective role of breastfeeding, describing a risk reduction of 15% [32].

The fundamental pillars of non-pharmacological treatment are lifestyle changes such as education, healthy eating, and physical activity [12,13,24].

Indeed, families are educated about lifestyle changes, such as the reduction of sugar and animal origin fat consumption and the increase of vegetable and fruit consumption, before beginning a personalized diet [15,23,24,31]. Furthermore, it is recommended that there is moderate daily physical activity between 30 and 60 min [15,24,30,33]. Moreover, it is of key importance to assure adequate sleep hygiene and duration since sleep deprivation represents a risk factor for obesity development [15]. Lastly, it must be investigated whether there is discrimination and bullying to prevent or cure social problems that could be associated with obesity [14].

Pharmacotherapy for weight loss in pediatrics is debated and represents a second-line approach. Actually, a limited number of anti-obesity medications have been approved for the pediatric population compared to adults. Pharmacological treatment is considered for subjects with severe obesity older than 10 years of age who have not responded to 1-year nutritional and lifestyle treatments, as well as for those with multisystem comorbidities [29].

Despite their therapeutic efficacy, drugs currently used for weight loss have many limitations because of their high incidence of side effects (between 3% and 44% depending on the drug used and different studies), prohibitive costs, and unpopularity due to historical problems associated with their use [34].

Surgical treatment is reserved for cases of resistance to weight loss and control of comorbidities after lifestyle changes and/or pharmacological treatment [15,24,30,33].

## 4. Micronutrients Deficiency in Childhood Obesity

Childhood obesity is an important risk factor for different adverse nutritional conditions. Among these, it should be considered also trace element deficiencies. Indeed, specific features of obesity or the bad habits typically associated with it can lead to deficiencies in different trace elements.

### 4.1. Iron Deficiency

Little evidence is available regarding the association between pediatric obesity and trace element deficiency, although obesity and iron deficiency in particular are two of the most common malnutrition conditions in the world [35]. Two recent meta-analyses demonstrated an association between childhood obesity and increased risk of iron deficiency: the first one involved only patients under the age of ten years [36], while the more recent and second one confirmed the presence of this association even in older patients [37].

Different assumptions can be made to explain this association. First of all, iron deficiency can be caused by poorer iron nutritional intake due to unhealthy eating habits (junk food, low fruit and vegetable consumption, and more processed food consumption) [37]. However, this hypothesis does not completely explain iron deficiency: indeed, few studies regarding iron deficiency in childhood obesity also included analysis of nutrient intake, and the association between the two conditions was confirmed even when including diet intake as a covariate [8,38].

Other mechanisms explaining iron deficiency in obesity are not fully understood. It is hypothesized that it can be due to increased iron requirements for elevated blood volume consequent to adipose tissue [39].

Furthermore, increased inflammation may contribute to iron deficiency by reducing iron absorption [39,40,41]. Indeed, obesity is linked to a state of persistent low-grade inflammation because of metabolic problems brought on by cells’ increased exposure to fatty acids [42]. Free fatty acids usually accumulate in ectopic places when the storage capacity of adipose tissue is exceeded by the amount of fatty acids present, and this exposure may cause cell malfunction, cell death, and inflammation [43].

Iron shortage and inflammation are related, since Hepcidin, which has a role both as a homeostatic regulator of systemic iron metabolism and as a mediator of host defense and inflammation, is required for its regulation [44,45,46]. Hepcidin is produced by the liver, which represents the primary site for its secretion in response to the detection of levels of both circulating iron and iron stores [46,47]. It prevents the intestinal absorption of iron and binds to ferroportin-1, promoting its internalization and degradation [47].

Proinflammatory cytokines, including interleukin (IL)-6, bone morphogenetic proteins, and iron overload increase the hepatic synthesis of Hepcidin. On the other hand, iron insufficiency, hypoxia, and inefficient erythropoiesis downregulate its production [48]. For this reason, inflammatory anemia, which is defined by decreased iron levels in the blood and increased cellular iron reserves, is associated with higher levels of hepcidin [49]. Therefore, it is reasonable to assume that the chronic low-grade inflammation associated with obesity can produce inflammatory cytokines, leading to the release of hepatic Hepcidin [40].

**Figure 2:**
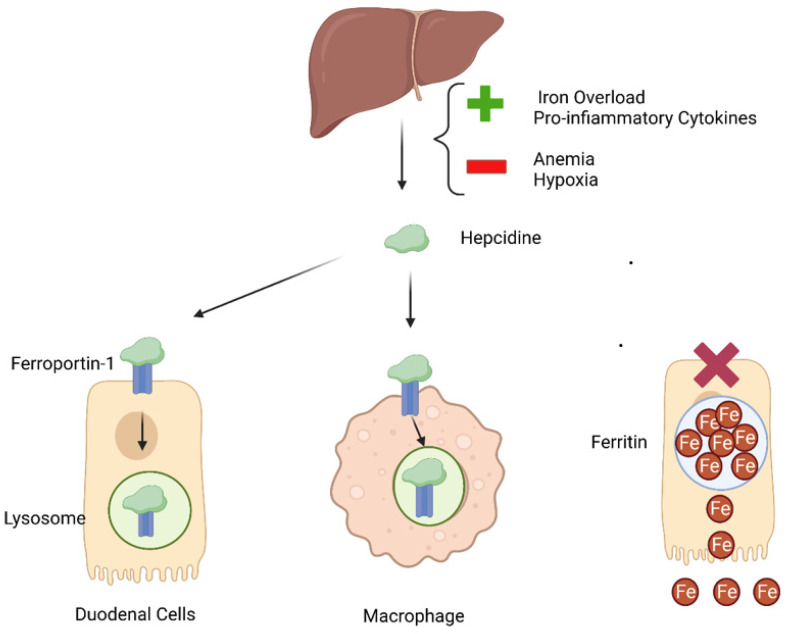
Inflammation effect on iron metabolism (created with BioRender.com (accessed on 15 March 2023)).

To further support this hypothesis, a study reported that overweight children had higher circulating Hepcidin and poorer iron status when compared to normal-weight children, despite similar dietary iron intake [50]. In conclusion, low-grade inflammation typical of the obesity condition causes an increased release of Hepcidine by the liver. Hepcidine binds to ferroportine expressed by different tissues, resulting in its internalization in the cell and degradation in the lysosome. This is especially significant for the duodenal mucosa, where iron is absorbed, and for macrophage cells, which are appointed to recycle iron from senescent or damaged erythrocytes [51]. Moreover, a small fraction of intracellular iron is maintained in the labile iron pool in the cell cytosol, while the majority is utilized in enzymes or sequestered in ferritin to prevent iron-mediated oxidative damage. Ferritin is upregulated by inflammation, with consequent increased iron stores in the cells [52]. Effects of inflammation on iron metabolism are shown in Figure 2.

Lastly, the synthesis and secretion of Hepcidin have also been documented in adipose tissue explants, but in vivo investigations did not support these results. Therefore, additional research is required to more clearly define the function of adipose tissue in systemic Hepcidin levels [53].

Two separate research studies found that oral iron supplementation therapy was ineffective at replenishing iron stores in children with obesity. This provided additional evidence on the crucial role of inflammation in iron deficiency in childhood obesity. According to both studies’ findings, blood Hepcidin was linked to hypoferremia and a worse outcome from oral iron therapy [54,55]. On the other hand, two studies showed that weight loss was linked to improved iron and inflammatory status in several groups of obese children [56,57].

Given that low-grade inflammation is a hallmark of obesity, it is critical to find indicators that may distinguish anemia caused by chronic disease from anemia due to an iron shortage in a patient with a chronic condition such as obesity. The gold standard for identifying iron deficiency and iron deficiency anemia is still a bone marrow test, but it is a painful, intrusive, and expensive procedure [58]. Serum iron, serum ferritin, soluble transferrin receptor, and transferrin saturation are common biomarkers of iron status that can be useful for the diagnosis of anemia [59]. When there is anemia, serum iron levels are always low. The serum ferritin level, which is indicated by a level of less than 30 g per liter, is the most sensitive and specific test used to detect iron deficiency. The strongest predictor is a ferritin level of less than 100 g/L, and a substantially higher cutoff ferritin level should be used to establish iron-deficiency anemia accompanied by inflammation. Less than 16% transferrin saturation levels signify an inadequate iron supply to maintain normal erythropoiesis [60]. Increased serum levels of the soluble transferrin receptor (s-TfR) are suspected in an iron deficiency condition [61]. Hepcidin levels can currently not be accurately measured [60]. In Table 2, the most important laboratory findings in different kinds of anemia are summarized.

Another possibility for a correct diagnosis to avoid mistakes due to inflammation or aging is to evaluate a complete blood count with reticulocyte hemoglobin content (CHr). Reticulocytes are markers of iron availability in iron-deficient erythropoiesis [59].

It is very important to identify and treat iron deficiency in children because of its consequences in pediatrics. Indeed, it has been demonstrated that iron deficiency has a negative effect on motor skills, cognition, and behavior that can persist in the long term [62].

### 4.2. Vitamins A, C, and E Deficiency

Children and adolescents with obesity have been found to have a higher risk of low vitamin A, vitamin E, and vitamin C concentrations than children and adolescents with normal weight [9,63]. Vitamins A, C, and E in particular have been demonstrated to be related to leptin concentration [64,65].

Low vitamin C concentration was linked to greater body fat, abdominal fat, and waist/height ratio, according to research by Garcia and colleagues on 197 Mexican children [64]. By a variety of different methods, including control of adipocyte lipolysis [66], an inflammatory response [67], and suppression of leptin levels [68], vitamin C may reduce adiposity. Although there are not many studies on the effectiveness of vitamin C supplementation, some data suggests that it may lower leptin levels in the blood and reduce body weight and adiposity in rats [69].

Similar to this, it was shown that vitamin E concentrations were negatively correlated with all obesity-related indicators [64]. These findings are in line with earlier research that linked obesity and reduced vitamin E concentrations [9,70]. As suggested in the animal model, vitamin E may play a function in leptin metabolism, which could account for this connection [71]. Lastly, vitamin A concentration was discovered to be favorably correlated with indices of obesity by Garcia and colleagues [64]. These findings were in stark contrast to earlier research that did not focus on children but indicated an inverse relationship between vitamin A concentration and weight, BMI, and hip circumference [72,73]. The association between vitamin A and adiposity may be different in those with a greater BMI and body fat content since the action of vitamin A on adipogenesis, specifically retinoic acid, appears to be dose-dependent [74].

Moreover, triglyceride and total cholesterol concentrations are positively correlated with vitamin A and E concentrations [64]. This might be a result of how vitamins A and E affect lipid metabolism. In more detail, the retinaldehyde dehydrogenase 1 enzyme helps vitamin A regulate lipid metabolism [75]. In order to avoid oxidative damage and preserve lipids from oxidation, vitamin E is important [76].

Regarding the supplementation of vitamin A in teenagers, no systematic reviews are available. Only a small number of studies on teenagers from Bangladesh [77], Kenya [78], and Indonesia [79] examined the effects of vitamin A supplementation, which significantly reduced anemia, but it is not clearly described how it affects body composition.

### 4.3. Vitamin B Deficiency

During childhood and adolescence, it is critical to maintain adequate folate and cobalamin status for cellular processes, notably nucleic acid synthesis, cell division, homeostasis of both amino acids and lipids, red blood cell formation, and nervous system myelination [80,81].

Their deficiency has been linked to several risks, such as failure to thrive, delayed psychomotor development, megaloblastic anemia [82,83], and, recently, poor event-free survival in cancer [84]. Moreover, low levels can cause DNA synthesis interference, cellular inflammation, elevated synthesis of fat, and hyperhomocysteinemia, an independent risk factor for insulin resistance and cardiovascular disease [85,86].

Studies on optimal serum concentrations in healthy European children are still scarce, and no universally accepted cut-offs have been established [87].

However, several studies have assessed that children with malnutrition related to overweight, obesity, and metabolic syndrome are at increased risk of mineral and vitamin deficiencies [88,89]. Hence, reduced vitamin B12 and folate serum levels may be caused by poor dietary content; short-term, repeated restricted diets; or increased requirements [11,89].

In a prospective and descriptive study, Hamiel et al. [9] demonstrated that Israeli children and adolescents with obesity (aged 6 to 19 years) had significantly lower vitamin B12 concentrations when compared to those with normal weight, and that 10% of children with obesity had low serum B12 concentrations. Obesity was associated with a greater than 4-fold risk for low cobalamin levels, and for each unit increase in BMI-standard deviation scores (SDS), there was a 24% increased risk of low serum cobalamin concentrations [11].

Additionally, in a randomized controlled trial conducted on adolescents with obesity (mean age of 13.4 years) with pre-diabetes and/or a clinical feature of insulin resistance, vitamin B12 status was found below or borderline in almost one-third of them [90].

Gunanti et al. also showed that vitamin B12 serum concentrations and folate levels in Mexican children (aged 8–15 years) were inversely associated with BMI and that higher serum concentrations of vitamin B12 were associated with a reduced obesity risk [91].

Accordingly, a recent prospective, longitudinal cohort study, based on a population aged between 2 months and 18 years, reported an inverse correlation between BMI-SDS with respect to both folate and cobalamin; the correlation between cobalamin, but not folate, and BMI-SDS strengthened with increasing age [87].

Healthy eating habits should be promoted to prevent obesity and its complications. Moreover, dietary treatment of children with obesity should focus not only on reducing dietary calories but also on choosing high-nutrient density foods [92].

Preclinical and clinical studies in rats and adults, respectively, showed that vitamin B12 and folic acid supplementation could reduce the risk of developing cardiac and cerebral vascular disease and may have positive effects on anthropometric and metabolic biomarkers of obesity, such as triglyceride, HDL-C, and insulin serum levels, HOMA-IR, and waist-to-hip ratio [86].

To date, a randomized, double-blind, placebo-controlled trial has shown that folic acid supplementation could reduce plasma homocysteine (tHcy) in children with obesity, especially in males and those with low serum folate levels [93]. Moreover, Peña et al. demonstrated that. Folic acid supplementation improved endothelial function in children with type 1 diabetes, increased folate status, and reduced tHcy in children with obesity without diabetes [94].

### 4.4. Vitamin D Deficiency

Vitamin D has many crucial roles in the human body: it is very important for calcium and phosphorus metabolism in the bone system, for immune functions, and some researchers found that it decreases the risk of chronic illnesses [95].

During the last few decades, a lot of studies were able to underline the prevalence of vitamin D deficiency in childhood and they found a significant association between childhood overweight and obesity with vitamin D deficiency (25-OH vitamin D < 20 ng/dL).

Turer et al. designed a cross-sectional study in the USA in which 12,292 children aged 6–18 years were included. This is one of the first studies that aimed to evaluate the vitamin D deficiency rate in children with obesity in the USA. It showed a significantly higher prevalence of vitamin D deficiency among children with overweight and obesity compared to those with healthy weight control, and severe obesity appeared to be particularly at risk; in fact, one in two children with severe obesity were vitamin D deficient, even after adjustments for cofounding factors [96]. Subsequent meta-analyses later confirmed vitamin D deficiency as a condition associated with obesity in young people [97,98].

The most likely explanation of this association appears to be the sequestration of fat-soluble vitamin D within the enlarged adipose mass in people with obesity [99,100]. Wortsman et al., in fact, highlighted that obesity does not affect the skin’s capacity to produce vitamin D3, but instead it may alter the vitamin D3 release from the skin into the circulation because of a more sequestered rate of cutaneously synthesized vitamin D3 by subcutaneous fat [99]. Leptin, which is overrepresented in populations with obesity, could also be responsible for this association. There is evidence in populations with obesity that leptin appears to activate a pathway that inhibits the renal enzyme responsible for the active form of vitamin D production [101]. In addition, obesity is mostly associated with a more sedentary lifestyle, usually associated with less sun exposure, and this association can also explain why children with obesity have lower circulating levels of vitamin D [10,102]. Eventually, except in some populations where fish, fish oil, and fish eggs are frequently consumed, children with obesity have even lower intakes of vitamin D, subsequently resulting from poor quality diets.

Some studies have investigated a possible association between ethnicity and vitamin D deficiency. Accordingly, the prevalence of vitamin D deficiency was significantly higher in Afro-American children with obesity compared with Caucasian subjects [96,102]. However, it is known that Afro-American individuals do have a reduced skin synthesis of vitamin D due to their darker skin pigmentation [103], and this association may also be attributable to the high rates of obesity in black children [104,105].

In the current literature, certain studies aim to assess whether there are differences in circulating vitamin D levels following oral supplementation in patients with obesity compared with control patients. In Wortsman et al.’s study, it was found that oral vitamin D supplementation at higher doses in the population with obesity is still required [99]. In this line, Harel et al.’s study also suggests higher doses of administration in those children with obesity who do not normalize their vitamin D circulating level after the initial course of treatment [10]. This is important because several studies have described the beneficial effects of vitamin D supplementation in the treatment of MetS-related diseases, such as lipid profile, insulin resistance and hyperglycemia, hypertension, and obesity [106,107]. There is limited evidence in children, and study results are quite controversial. A recent meta-analysis, which included eleven randomized-control trials based on an adult population, demonstrated that vitamin D supplementation could contribute to reduced BMI and waist circumference but not to weight loss [108]. On the contrary, individuals aged 6–14 years showed no significant effects of vitamin D supplementation on BMI, waist circumference, waist-to-hip ratio, and percentage of fat tissue [109,110,111].

An adequate vitamin D status is important during the entire pediatric age, above all to guarantee bone health and avoid nutritional rickets, but it is seen that its supplementation could be useful in children with obesity too. However, to date, population-wide vitamin D deficiency screening in healthy individuals is not recommended, and 25(OH)D evaluation should be reserved for subjects at vitamin D deficiency risk [112].

### 4.5. Zinc and Copper Deficiency

Micronutrient deficiencies are extremely widespread all over the world and have many consequences for public health [113]. Metallic elements have fundamental biological functions; their deficiencies may cause different diseases and disorders [12] and may be related to obesity and overweight [7].

Multiple studies have shown that trace element levels could be deficient in children with obesity [114]. Despite that, the relationship between serum copper (Cu) and zinc (Zn) concentrations and overweight/obesity is still controversial and needs to be further investigated. Zn is an essential micronutrient with an important biological role: it contributes to metabolic and endocrine regulation, immune response, and multiple biochemical reactions [12]. Cu plays an important role in preventing superoxide damage with its function in copper/zinc superoxide dismutase (Cu/Zn SOD), which helps to reduce the obesity risk [115]. Controversially, an excessive level of serum Cu can damage the oxidant/antioxidant system, causing an increase in reactive oxygen species (ROS) and reactive nitrogen species (RNS) in the organism [116].

Cu level disorders may also affect Zn metabolism, leading to an increase in oxidative stress and the inflammatory response, both of which seem to have a key role in obesity pathogenesis [116]. Several studies have analyzed the relationship between serum Cu and overweight/obesity; some of those found a significantly higher serum Cu level in children with obesity than in healthy controls [12,114,117,118,119], whilst others observed no relationship [120,121]. Interestingly, Lima et al. [117] reported a higher concentration of serum Cu in individuals with obesity but no difference in Cu erythrocyte concentration between children with obesity and healthy weight controls. Gu et al. [115] reported that the serum Cu level was higher in children and adults with obesity without any significant difference in serum Cu between the overweight and control groups of both populations. Their results suggest that a higher serum Cu concentration might be associated with an increased obesity risk [115]. Jacksic et al. [120] observed a strongly positive correlation between serum Cu level and C-reactive protein, which means that there may be an association between childhood obesity and oxidative stress and inflammation, even if no relationship between Cu and Zn concentrations and obesity was found.

On the other hand, certain studies analyzed the relationship between serum Zn and childhood obesity, with disputable results [12]. Some studies reported that Zn levels were lower in children with overweight/obesity [12,114]. DiMartino et al. [122] found that in the study population, BMI was significantly higher compared to the control group, and serum Zn concentration was significantly lower in the same groups. Similar findings were reported by Marreiro et al. [123] and Garcia et al. [64], which observed higher insulin resistance in children with low Zn concentration, which is related to an increased obesity risk.

It was also reported that children with overweight/obesity have significantly lower salivary zinc levels and a higher caries risk [124].

However, Weisstaub et al. [125] observed no significant relationships between serum Zn concentration and body weight in children. Cozzolino et al. [123] and Jacksic et al. [120] reported similar results analyzing the relationship between Zn nutritional status and childhood obesity.

## 5. Conclusions

In conclusion, obesity can be associated with multiple micronutrient deficiencies due to different mechanisms. The more relevant deficits and their causes are summarized in Table 3.

**Figure 3:**
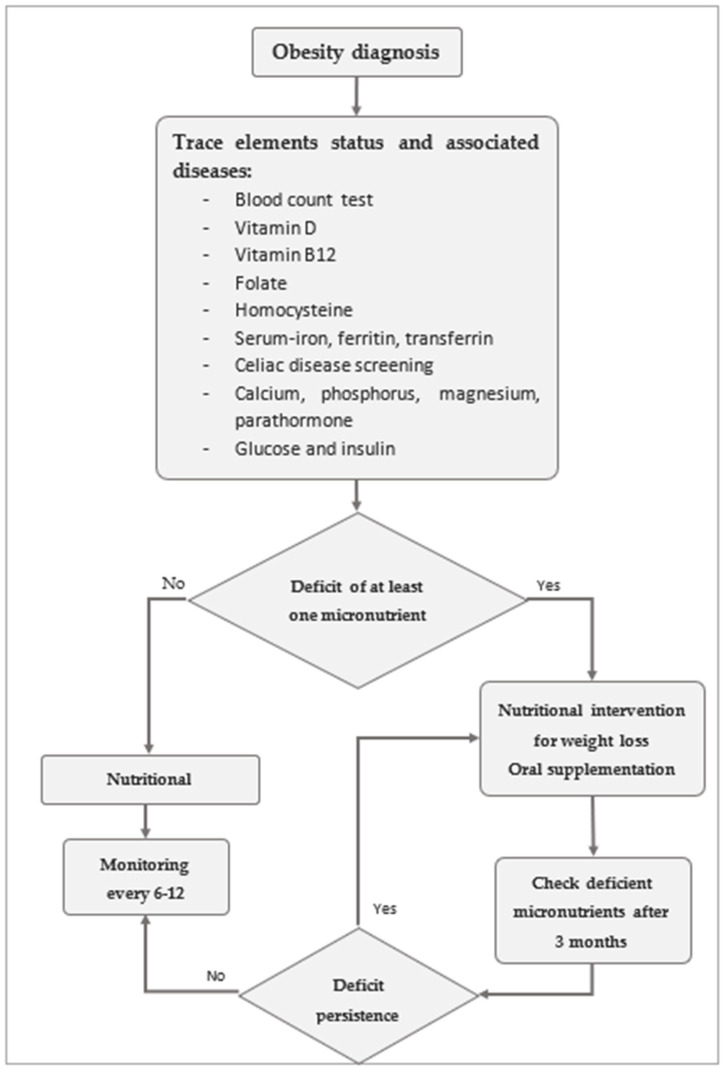
Micronutrient monitoring process.

As can be inferred, deficits are common in patients with obesity, and it is of key importance to identify and correct them to prevent possible complications. In Figure 3, a flowchart is proposed for the early diagnosis and monitoring of micronutrient deficiency in the patient with obesity to limit adverse effects related to malnutrition.

It is important to underline that obesity is a multifactorial disease. Therefore, in addition to overnutrition, ethnicity and genetic polymorphisms of the metabolic pathway, in which micronutrients are involved, can play a vital role in gaining fatty tissue. Knowing that, correct nutritional strategies can be personalized in order to optimize the maintenance of a correct weight and nutritional status.

Unfortunately, only a few studies are available regarding the efficacy of oral supplementation or weight loss for treating them; thus, continuous nutritional monitoring is necessary.

## Figures and Tables

**Table 1:** Obesity and overweight diagnostic criteria in children.

	Age		
	0–2 years	2–5 years	5–18 years
Index	Weight-to-lenght ratio	Body mass index	Body mass index
Reference	WHO 2006	WHO 2006	WHO 2007
>85th percentile *	Overweight risk	Overweight risk	Overweight
>97th percentile *	Overweight	Overweight	Obesity
>99th percentile *	Obesity	Obesity	Severe obesity

* The 85th, 97th, and 99th percentiles’ approximate z-scores of +1, +2, and +3, respectively. WHO—World Health Organization.

**Table 2:** Differential diagnosis of anemia.

	Iron Deficiency Anemia	Chronic Disease Anemia	Iron Deficiency Anemia in Chronic Disease
Serum Iron	Decreased	Decreased	Decreased
Ferritin	Decreased	Increased	Often Increased
s-TfR	Increased	Decreased	Increased

s-TfR—Soluble Transferrin Receptor.

**Table 3:** Possible mechanisms of deficiency.

Deficiency	Possible Mechanisms of Deficiency
Iron	Poor nutritional intake Increased iron requirements for elevated blood volume for increased adipose mass Reduced iron absorption because of enhanced inflammation
Folic Acid	Poor nutritional intake Increased requirements
Vitamin B12	Poor nutritional intake Increased requirements
Vitamin D	Sequestration in enlarged adipose mass Reduced release from skin Leptin mediated inhibition of renal enzyme responsible for active form of vitamin D

## Data Availability

Not applicable.
